# Mushrooms as Potential Sources of Active Metabolites and Medicines

**DOI:** 10.3389/fmicb.2022.837266

**Published:** 2022-04-26

**Authors:** Anne Bhambri, Malay Srivastava, Vivek G. Mahale, Sushma Mahale, Santosh Kumar Karn

**Affiliations:** ^1^Department of Biochemistry and Biotechnology, Sardar Bhagwan Singh University, Dehradun, India; ^2^M/s Xcel Life Sciences, Fremont, CA, United States

**Keywords:** mushroom, metabolites, medicine, ITS sequence, β-glucan, terpenoids, unknown metabolites, polyketides etc

## Abstract

**Background:**

Mushrooms exist as an integral and vital component of the ecosystem and are very precious fungi. Mushrooms have been traditionally used in herbal medicines for many centuries.

**Scope and Approach:**

There are a variety of medicinal mushrooms mentioned in the current work such as *Agaricus, Amanita, Calocybe, Cantharellus, Cordyceps, Coprinus, Cortinarius, Ganoderma, Grifola, Huitlacoche, Hydnum, Lentinus, Morchella, Pleurotus, Rigidoporus, Tremella, Trametes* sp., etc., which play a vital role in various diseases because of several metabolic components and nutritional values. Medicinal mushrooms can be identified morphologically on the basis of their size, color (white, black, yellow, brown, cream, pink and purple-brown, etc.), chemical reactions, consistency of the stalk and cap, mode of attachment of the gills to the stalk, and spore color and mass, and further identified at a molecular level by Internal Transcribed Spacer (ITS) regions of gene sequencing. There are also other methods that have recently begun to be used for the identification of mushrooms such as high-pressure liquid chromatography (HPLC), nuclear magnetic resonance spectroscopy (NMR), microscopy, thin-layer chromatography (TLC), DNA sequencing, gas chromatography-mass spectrometry (GC-MS), chemical finger printing, ultra-performance liquid chromatography (UPLC), fourier transform infrared spectroscopy (FTIR), liquid chromatography quadrupole time-of-flight mass spectrometry (LCMS-TOF) and high-performance thin-layer chromatography (HPTLC). Lately, the matrix-assisted laser desorption ionization-time of flight mass spectrometry (MALDI-TOF MS) technique is also used for the identification of fungi.

**Key Finding and Conclusion:**

Medicinal mushrooms possess various biological activities like anti-oxidant, anti-cancer, anti-inflammatory, anti-aging, anti-tumor, anti-viral, anti-parasitic, anti-microbial, hepatoprotective, anti-HIV, anti-diabetic, and many others that will be mentioned in this article. This manuscript will provide future direction, action mechanisms, applications, and the recent collective information of medicinal mushrooms. In addition to many unknown metabolites and patented active metabolites are also included.

## Highlights

- This manuscript highlights the medicinal mushroom species and their active metabolites.
- Detail of active metabolite and action mechanisms are mentioned.
- Recent technologies used for the identification of mushroom species are described.
- Active metabolite roles in various diseases are included.
- Unknown active metabolites and patented metabolites are also incorporated.

## Introduction

Human beings have cultivated and consumed mushrooms as a kind of macro fungi for centuries, due to their attractive characteristics such as their ease of cultivation, and their multiple functional activities (Roupas et al., 2012; Kalac, 2016; Phat et al., 2016; Roncero-Ramos and Delgado-Andrade, 2017). Macro fungi are economically essential because of their importance in medicine, biocontrol, food, and the biological, chemical, and other industries. These macro fungi differ in their uses as medicines and food and several other species form mycorrhizal associations and function as decomposers (Meena et al., 2020).

Fungi that belong to several taxonomic groups which produce conspicuous sporocarps are called macro fungi – these include truffles, gilled fungi, stink fungi, jelly fungi, birds’ nest, coral fungi, puffballs, and bracket fungi (Enow, 2013). These mushrooms live as saprophytes. In the decomposition process, all types of mushrooms are essential due to their ability to degrade cellulose as well as other polymers. Large fungi form large fruitifications that can be visible without the help of a microscope and these include Ascomycota and Basidiomycota having large spore-bearing structures (Al-Thani, 2010). In nature, mushrooms are widespread as well as the earliest form of fungi known to human beings (Okhuoya et al., 2010; Meena et al., 2020).

Mushrooms are seasonal macro fungi mainly found in the rainy season or when the snow melts, and form macroscopic fruiting bodies. Mushrooms can be leathery or woody, fleshy, or sub-fleshy. They bear their fertile surface on the lining of the tubes or on the lamellae and open out by means of pores. Polypores and Boletes are the tube-bearing poroid membranes whereas Agarics are the lamellate members (Deshmukh, 2004). Humans have consumed wild mushrooms from ancient times, with likely considering them a delicacy and also due to their pleasing flavor (Das, 2010). Edible mushrooms are considered to be devoid of undesirable effects and have medicinal values (Barros et al., 2008; Martinez-Medina et al., 2021).

Mushrooms are rich in essential nutrients and have rich nutritional values with high contents dietary of fibers, significant contents of vitamins (B_1_, B_2_, B_12_, C, D, and E), mineral substances, trace elements, a high quality of proteins including important content of essential amino acids. They may however be limited in the cystine, methionine, and sulfur-containing amino acids, carbohydrates, and fats but with excellent important fatty acids content, low or no calories and cholesterol, and antioxidants, and are known as host defense potentiators (Okwulchie and Odunze, 2004; Agrahar- Murugkar and Subbulakshmi, 2005; Heleno et al., 2010; Wani et al., 2010; Niazi and Ghafoor, 2021).

Different mushrooms species are rich in ash (7–17%), fiber (16–20%), protein (30–48%), fat (1–4%), and carbohydrate (125–40%), etc. (Manikandan, 2011). Mushrooms also have bioactive nutraceuticals such as polysaccharides (Ruthes et al., 2016), lectins (Singh et al., 2014), phenolic compounds (Heleno et al., 2015a), glycoproteins (Kumar, 2015), ergosterols (Barreira et al., 2014), unsaturated fatty acids (Tel-Cayan et al., 2017), and tocopherols (Khatua et al., 2013). These mushrooms also contain four influential nutrients like ergothioneine, glutathione, vitamin D, and selenium which serve to alleviate oxidative tension as well as an antioxidant property (CNN Health, 2018).

**FIGURE 1:**
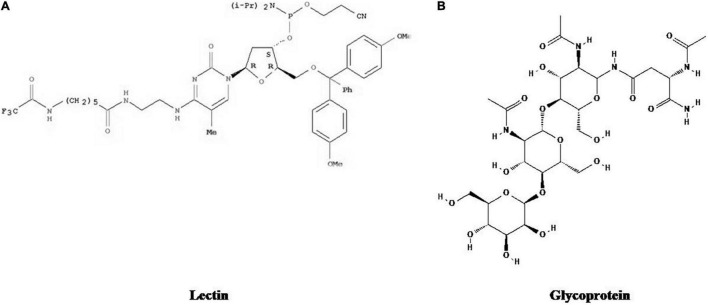
Structures of active metabolites of mushroom: **(A)** Lectins (Butt and Khan, 2019) **(B)** Glycoprotein (National Center for Biotechnology Information [NCBI], 2022b).

**FIGURE 2:**
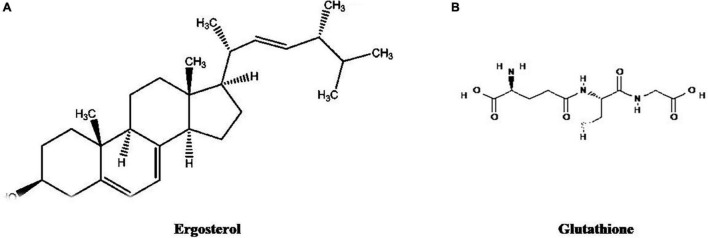
Structures of active metabolites of mushroom: **(A)** Ergosterol (Shao et al., 2010) **(B)** Glutathione (National Center for Biotechnology Information [NCBI], 2022a).

**FIGURE 3:**
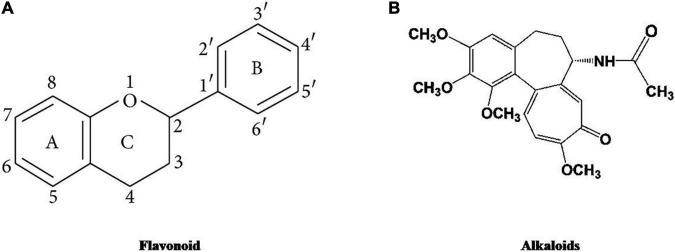
Structures of active metabolites of mushroom: **(A)** Flavonoid (Kumar and Pandey, 2013) **(B)** Alkaloids (International Union of Pure and Applied Chemistry, 1995).

**FIGURE 4:**
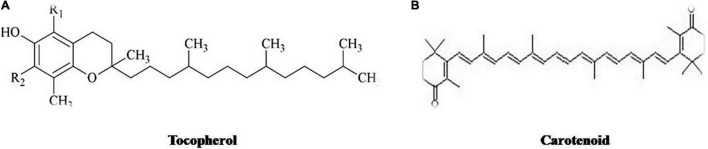
Structures of active metabolites of mushroom: **(A)** Tocopherol (Bauerova et al., 2019) **(B)** Carotenoid (Eldahshan and Singab, 2013).

**FIGURE 5:**
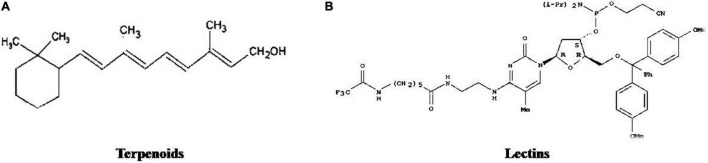
Structures of active metabolites of mushroom: **(A)** Terpenoids (Yadava et al., 2014) **(B)** Lectins (Butt and Khan, 2019).

There are various bioactive compounds found in cultured broth, mycelium, and fruiting bodies such as volatile oils, flavonoids, alkaloids, ascorbic and organic acids, fats, polysaccharides, tocopherols, glycosides, minerals, proteins, carotenoids, terpenoids, lectins, enzymes, phenolics, and folates (Venturella et al., 2021). Figures 1–5 represents the structures of active metabolites of mushroom. Of which, polysaccharides are extremely important for modern medicine. Moreover, β-glucan is not a versatile and well-known metabolite, having a wide spectrum of biological activity (Chang and Wasser, 2012; Patel and Goyal, 2012; Finimundy et al., 2013; Kiss et al., 2021). Mushrooms also have a high percentage of water (93–95%) and also contain valuable minerals like copper, phosphorus, iron, potassium, and calcium. Due to their high protein and low-calorie values, they are recommended to heart patients and their essential amino acids are required by adults to keep healthy (Koyyalamudi et al., 2009).

**FIGURE 6:**
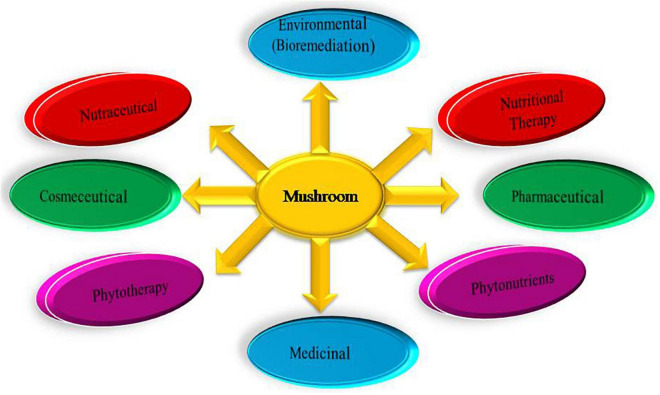
Representing the wide applications of mushroom.

Mushroom has a high concentration of tryptophan and lysine as compared to cysteine and methionine. They are also a good source of ascorbic acid and pantothenic acid as well as an excellent source of nicotinic acid and riboflavin (Ukpebor et al., 2007; Seo and Choi, 2021). Mushroom is also an ideal food for persons who wants to reduce excess fat as well as for diabetic patients due to the absence of starch. The wide applications of mushrooms are represented in in Figure 6.

This manuscript deals with medicinal mushrooms and their nutraceutical as well as bioactive compounds. There are various active metabolites such as polysaccharides, proteins, and peptides, terpenes, phenolic compounds, polyunsaturated fatty acids, carbohydrates and lipids are mentioned in this manuscript. There are several other unknown metabolites that are present in mushroom and can be beneficial for human health. This manuscript also deals with the medicinal mushrooms that are found in India and other countries and also mentions various identification techniques. Furthermore, omic approaches and the current status of each medicinal mushroom propagation have been mentioned.

## Nutraceuticals and Bioactive Compounds

For thousands of years, mushrooms have been used in folk medicines. Some of them are nutraceuticals whereas others can produce bioactive compounds (Elmastas et al., 2007; Ribeiro et al., 2007). Huge numbers of mushroom species are not only edible and nutritious, but also possess toxic and medicinal qualities. Mushrooms are not only used as healthy vegetables that are rich in proteins but also a source of biologically active compounds having medicinal values. Uses such as hepatoprotective agents, immune-potentiating, complementary medicine or dietary supplements for anticancer and antiviral purposes (Olawale and Muhammed, 2017).

In recent years, there have been many studies focusing on the therapeutic and health benefits of the specific bioactive nutraceuticals of mushrooms such as antitumor, antibacterial, antioxidant, antiallergic, immunomodulating, cardiovascular protector, detoxification, antiviral, antifungal, antiparasitic, immune function enhancement, antioxidant, anti-inflammation, hepatoprotective, hypolipidemic, antithrombotic, antidiabetic, hypotensive, antiproliferative, anti-HIV, hypo-cholesterolemic, anticancer, cytotoxic, and anticoagulant activities (Wasser and Weis, 1999; Lindequist et al., 2005a; Ajith and Janardhanan, 2007; Yu et al., 2009; Zhang et al., 2011; Chang and Wasser, 2012; Finimundy et al., 2013; Rathore et al., 2017). Additionally, mushrooms also have the ability to attenuate the health hazards induced by obesity and the impaired functions caused by aging (Yahaya et al., 2014; Sayeed et al., 2015; Thangthaeng et al., 2015; Ghosh, 2016; Persson, 2016) (Table 1).

**TABLE 1:** Showing mushroom metabolites, mechanism and their applications.

S.No.	Name of Medicinal Mushroom	Metabolite components	Treatment/Applications	Mechanism	References
**1.**	***Trametes***	Carbohydrates, Lipids, Proteins, Flavanols, Bioflavonoids, Iso-flavonoids, Flavones, Flavanone, Ergosterol, β-sitosterol, Stigmast-5-en-3-ol, Hydroxy methylquinoline, Sesquiterpene, Coriolin and De-oxycoriolic acid	Antibacterial, Anticancer, Insecticidal, Antioxidant, Anti-proliferative, Anticoagulant, Antifungal, Antidiabetic, Hepatoprotective, Antiparasitic, Antiviral, Anti-inflammatory, Upper respiratory, Digestive, Urinary Tract Infections and Chronic Hepatitis	Immunostimulant, Activation of macrophage and Natural killer cell cytotoxicity.	Hobbs, 2004; Habtemariam, 2020
**2.**	***Agaricus***	Fatty acids, Phenolics, Amino acids, Sugar and Polyols, Organic acids, Lectins, Unsaturated fatty acids (linoleic and linolenic acids), Sterols, Phenolic, Indole compounds and Nutraceuticals	Liver Disease, Cancer, Digestive Problems, High cholesterol, Type 2 Diabetes, Arteriosclerosis, Bloodstream Disorders, Heart Disease, Osteoporosis and Stomach Ulcers	Inhibit cell proliferation of some cancer cell line, Antioxidant activities, Anti-inflammatory	O’Gorman et al., 2011; Usman et al., 2021
**3.**	***Ganoderma***	Triterpenoids, Polysaccharides, Proteins and Peptides, Terpenoids, Phenols, Glycoproteins, Triterpenes, Amino acids (Lysine and Leucine), Ganodermic acids, Nucleotides and their derivatives, Peptidoglycans and Steroids	Diabetes, Infections, Cancer, Immune System Disorders, Hepatoprotection, Bacterostasis, Bronchitis, Gastric Ulcer, Hepatopathy, Asthma, Insomnia, Chronic Hepatitis, Nephritis, Arthritis, Hypertension, Weakness, Fatigue, Cough, Anti-atherosclerosis, Anti-oxidant, Anti-HIV, Nephroprotective, Anti-tumor, Anti-hepatotoxic, Cardiovascular, respiratory Properties. It also decreases the level of blood pressure, Inhibition of platelet aggregation as well asblood cholesterol, Anti-inflammatory, Analgesic, Chemopreventive, Chemo &Radio protective, Sleep promoting, Antibacterial, Antiviral, Hypolipidemic, Anti-fibrotic, Anti-&rogenic, Anti-angiogenic, Anti-herpetic, Radical-scavenging, Anti-aging, Hypoglycemic, Estrogenic activities.	Immunomodulator (interleukin – 12 production), Nitric oxide synthase activation	Sanodiya et al., 2009; Paliya et al., 2014
**4.**	***Hydnum***	Polyphenolic compounds such as Phenolic acids, Flavonoids, Hydroxybenzoic acids, Lignans, Tannins, Stilbenes, Oxidized polyphenols, Ferulic acid, Sarcodonin A, Savronine B and Quercetin	Antioxidant, Antimicrobial, Genotoxic, Protective against chemotherapeutics, Cytotoxic activity against a variety of tumor cells type, mainly colon adenocarcima cells	Synthesis of nerve growth factor	Tubic et al., 2009
**5.**	***Coprinus***	Carbohydrates, Dietary fibres, Proteins and Phenolic compounds.	Regulate the blood glucose level, Hypoglycemic and has Antitumor, Antioxidative, Hypolipidemic, Antibacterial as well as Immunomodulation effects	Regulate antioxidative homeostasis	Stilinović et al., 2020
**6.**	***Morchella***	Sugars, Organic acids, Flavonoids, Triglycerides, Free fatty acids and Sterols	Anti-inflammatory as well as Antitumor activity against both ascites as well as solid tumours of ethanolic extracts, High antioxidant activity	Immunomodulator, Increase the cytotoxic effect	Dissanayake et al., 2021
**7.**	***Cantharellus***	Phenolic compounds, Terpenes, Steroids, Lectins, Polysaccharides, Proteins, Phenolic compounds, Flavonoids, and Tannins	Excellent Antihyperglycemic, Antioxidant, Wound healing, Antimicrobial, Iron-chelation, Cytotoxicity, Anti-hypoxic, Anti-inflammatory activities	Causing Cytotoxicity against angiotensins converting enzyme	Palacios et al., 2011; Kozarski et al., 2015
**8.**	***Amanita***	Ibotenic acid, Muscazone and Muscimol	Antitumor, Pesticidal, Cytotoxic, Antioxidant, Anticancer, Antibacterial, Acetylcholinesterase, Esterolytic, Antiviral, Anti-, larvicidal, Antifungal, Anti-inflammatory properties	Induces Cascade dependent apoptosis	Cadet and Bolla, 2007; Pišlar et al., 2016
**9.**	***Cortinarius***	Amino acids, Orellanine	Antioxidant, Antihyperglycemic, Wound healing, Antimicrobial, Iron-chelation, Cytotoxicity, Anti-hypoxic, Anti-acid inflammatory	Inhibit protein synthesis	Meena et al., 2020
**10.**	***Tremella***	Fatty acids, Proteins, Enzymes, Polysaccharides, Phenols, Flavonoid, Dietary fiber and Trace elements.	Fight cancer, Combat obesity, Anti-aging, Lower cholesterol, Protect nerves and Anti-inflammatory.	Enzyme inhibition	Yang et al., 2019
**11.**	***Rigidoporus***	Anthraquinones, Alkaloids, Tannins, Saponins, Phlobatannins, Steroids, Flavonoids, Terpenoids and Cardiac glycosides.	Mitogenic activity, Anti-hepatitis B surface antigen effect, Plasma clotting activity, Activation of alternative pathway complement, Tumour suppressive effects	Exact mechanism is unknown, Antioxidant activities, Anti-inflammatory	Cheng et al., 2009
**12.**	***Grifola***	Polysaccharide (glucans), Sesquiterpenes, Glycoproteins etc.	Antitumor, Anti-inflammation, Immunomodulation, Antivirus, Antidiabetic, Immune-enhancing, Anti-hypertensive, Antioxidation, Non-alcoholic fatty liver disease, Hyperlipidemia and Hyperglycemia.	Immunomodulator	Su et al., 2020
**13.**	***Lentinus***	Phenolic compounds, Polysaccharides, Terpenoids, Sterols and Lipids	Fungal infection, Bronchial inflammation, Hyperlipidemia, Hepatitis, Cancer, Depressed immune function, Heart disease, Infectious disease, Flu and Cold, Environmental allergies, Urinary inconsistencies, Hypertension, Diabetes	Inhibitory effect on interleukin- 1β, tumour necrosis factor α	Resurreccion et al., 2016
**14.**	***Pleurotus***	Terpenoids, Steroidal glycosidase, Tannin	Anti-cholestrolic, Anticancer, Antiviral, Antidiabetic, Antioxidant, Eye health, Antibacterial and Antiarthritic.	Hypocholesterolemic, antherogenesis inhibition	Deepalakshmi and Mirunalini, 2014
**15.**	***Calocybe***	Ascorbic acid, Lipids, Riboflavin, Amino acids, Pyridoxine, Vitamins, Biotin, Low fat, Nicotinic acid, Proteins, Minerals (arsenic, zinc, potassium, manganese, calcium, phosphorus, magnesium, iron and sodium), Fibers	Reducing the triglycerides and total plasma cholesterol level and consequently decreases the chance of cardiovascular, artery and atherosclerosis related disorders, like neurodegenerative diseases, Anti-carcinogenesis, Anti-ageing, Anti-obesity, Cardiovascular disease, Anti-infectious, prevent from physical injury, Anti-tumour also helps to reduce the risk of breast cancer	Immunomodulator, immunegenerator	Sharma et al., 2013
**16.**	***Huitlacoche***	Contain Anthocyanins and Phenolic compounds which are Phytochemicals, Phytosterol, Polyphenols, Flavonoids, Proteins, Amino acids, Glutamic acid, Lysine, Serine, Aspartic acid, Glycine, Total carbohydrates, Arabinose, Mannose, Galactose, Xylose, Glucitol, Mannitol, Glycerol, Heteroglycans, Dietary fiber and Homoglycans	Antitumoral, Antimutagenic, Immunomodulating, Antiatherogenic, Hyperlipidemic, Hypoglycemic, Anti-inflammatory as well as Various other health promoting activities	Exact mechanism is unknown, Antioxidant activities.	Valverde et al., 2015
**17.**	***Cordyceps***	Alkaloids, Amino acids, Proteins, Carbohydrates, Flavonoids, Phenols, Gums, Mucilages, Saponins, Cordycepic acid and Cordycepin substances	Improved reproductive activity, Blood sugar metabolism, Effects of enhanced utilization of oxygen and Production of ATP. This mushroom protects the organs from kidney, liver as well as heart diseases.	Immunomodulating effects, Enhancement of neuromuscular activity, Endurance enhancing activity.	Rakhee et al., 2016

Mushrooms can cure wounds, stress, rheumatoid arthritis, asthma, diabetes, diaphoretic, liver disease, epilepsy, skin diseases, heart ailments, insomnia, allergies, cholesterol reduction, cholera besides intermittent fever, cold, gall bladder diseases, diarrhea, dysentery, anesthesia and also used as vermicides (Bahl, 1983). It can also cure heart, cancer, and nervous problems.

Clinically essential as well as well recognized drugs of medicinal mushroom and also the used drugs such as progesterone, morphine, aspirin, vinblastine, digitoxin, taxol, cortisone, vincristine as well as various others derived directly or indirectly from the higher plants. Patented products of active metabolites are mentioned in Table 2. Dasgupta and Acharya (2019) and Azeem et al. (2020) demonstrated that the poisonous mushroom *Neonothopanus nimbi* has aurisin A and aurisin K, which are effective against the *Mycobacterium tuberculosis* as well as *Plasmodium falciparum* (Ajith and Janardhanan, 2007). The important effect of pharmacology as well as the physiological properties of mushrooms are the regulation of biorhythm, cure of several diseases, bioregulation (immune enhancement), improvement as well as prevention from life-threatening diseases including heart diseases, cancer, and cerebral stroke, and also the maintenance of homeostasis (Wasser and Weis, 1999).

**TABLE 2:** Showing patented products of medicinal mushroom.

S.No.	Claimed Product/Extract Name	Activity Claimed	Patent Application No.	Inventors
**1.**	Process for producing, methods and compositions of glucuronoxylomannan as nutriceutical agent from higher basidiomycetes mushroom	Control hyperglycaemia	US 6383799	Wasser and Reshetnikov (2002)
**2.**	Anti-aging/menopause symptoms relief using *Ganoderma lucidum* spores	Anti-ageing	US 6908614	Chung and Tong (2005)
**3.**	Antimutagenic effects of *Ganodermalucidum* spores	Antimutagenic	US 7087233	Chung and Tong (2006)
**4.**	Glycoprotein with antidiabetic, antihypertensive, anti-obesity and antihyperlipidemic effects from *Grifolafrondosa*, etc.	Antidiabetic	US 7214778	Zhuang et al. (2007)
**5.**	Mushroom extracts having anticancer activity	Anticancerous	US 7258862	Mahajna et al. (2007)
**6.**	Phytonutrient compositions prepared from *Agaricusbisporus, Lentinula edodes, Pleurotusostreatus* and *Grifola frondose*	Treatment of neurodegenerative diseases and radiation damage	WO2007US63984 20070314	Beelman et al. (2007)
**7.**	Food supplement prepared from *Grifolafrondosa, Pleurotuseryngii, Hericiumerinaceus*	Reducing blood sugar and regulating blood lipid levels	CN 101292726 A 20081029	Liu et al. (2008)
**8.**	Mushroom extract from *Hericiumerinaceus*	Anti-dementia substance inhibits the neuronal toxicity of amyloid beta-peptide (Aβ) and induce the synthesis of nerve growth factor (NGF)	US2009274720 (A1)	Zhuang et al. (2009)
**9.**	Basidiomycetes, Basidiomycetes extract composition, health foods and immunopotentiators	Immune Function	US 7517682	Watanabe (2009)
**11.**	Ganoderic acid T-amide derivative TLTO-A	Antitumor agent for inhibiting cancer cells and induce the apoptosis of tumor cells	CN102219822	Zhong et al. (2011)
**12.**	Method of eritadenine production in liquid phase fermentation of *Lentinus edodes*	Blood cholesterol reducing therapeutic agent	United States Patent 8053217	Berglund et al. (2011)
**13.**	Mushroom extracts from *Agaricus, Hericiumerinaceum*, and *Hypsizigusmarmoreus* as insulin secretion stimulators and health foods for prevention and therapy of diabetes mellitus	Antidiabetic	JP 2012077004A	Takeshi et al. (2012)
**14.**	Terpenoid spiroketal compound from *Agaricussubrufescens* and related *Agaricus* sp.	Therapeutic potential on disease having Liver X Receptor (LXR) agonists activity	EP 2 468 253(A1)	Grothe et al. (2012)
**16.**	Antiviral and antibacterial activity from medicinal mushrooms	Antiviral, Antibacterial	US 8765138	Stamets (2014)
**17.**	Anti-cancer combination treatment and kit-of-parts	Anticancerous	US 9072776	Kristiansen (2015)
**19.**	Method to prepare *Ganoderma lucidum* polysaccharides possessing anti-obesity properties and uses there of	anti-obesity	US 9758595	Ko et al. (2017)
**20.**	Antiviral products	helps in the reduction of pathogenicviruses and treating viral infections, particularly viruses which afflict animals but not limited to pigs, birds, humans, bats and bee results in the reduction of diseases that causes viruses and their infectivity or pathogenicity in both the environment as well as in animal host.	US 9,931,316B2	Stamets (2018)

The production of secondary metabolites as the families of compounds having the same biosynthetic pathway such as non-ribosomal terpenoids or peptides. Anke (2020) proposed a theory for the existence of secondary metabolism. Consequently, secondary metabolism plays a vital role in the development of new chemical tools and also for the interaction with a changing environment during evolution (Anke, 2020). Mushroom species are divided into the edible mushrooms with representatives of *Agaricus bisporus, Auricularia auricula, Pleurotus ostreatus, Lentinula edodes*, and *Flammulina velutipes*, and medicinal mushrooms such as *Poria cocos, Ganoderma lucidum, and Cordyceps sinensis.*

Another, Psychedelic mushrooms—also known as magic mushrooms—are not the conventional type of mushroom, their extract delivering psychedelic effects which other mushrooms seldom do. Nevertheless, these magic mushrooms are not usually utilized in the fermented mushroom pills as well as the mushroom products manufactured by mushroom manufacturers. The US Food and Drug Administration (FDA) has banned magic mushrooms. No organic certified mushroom product has been eligible to contain magic mushrooms as an ingredient under the USDA (United States Department of Agriculture) (Dasgupta, 2019).

Globally, China was the first country to cultivate mushrooms and has the highest total production of mushrooms, followed by other countries such as Poland, the United States, The Netherlands, and Italy. Societies like Mexico, the ancient Greeks, Chinese, Romans, and Egyptians appreciated and treated mushrooms as medicines for a very long time (Feeney et al., 2014). Mushroom plays an important role in the food industry, biodegradation, in medicine, and in human welfare in general (Ozturk et al., 2003). These edible mushrooms are either harvested and cultivated under suitable conditions with rigorous quality control on palatability, shape, size, and tenderness, or collected directly from nature (Rahi and Malik, 2016). According to the derivation of mushroom names, edible mushrooms are categorized according to their morphology, texture, taste, habitat, and growth habits (Oso, 1975). Beside these edible mushrooms, there are also some non-edible or poisonous mushrooms like *Celtis zenkeri, Coprinus africans, Phallus industiatus, Coprinus ephemerus, Phallus aurantiacus, Phallus rubicundus*, and *Mutinus bambustnus*, etc.

## Mushroom as Medicine

An alternative source of new antimicrobial compounds might also be mushrooms, especially secondary metabolites which induce benzoic acid derivatives, quinolones, steroids, terpenes, anthraquinones, etc., but also some primary metabolites such as proteins, oxalic acid, and peptides (Alves et al., 2012); Zhong and Xiao (2009) demonstrated that the higher mushroom produces an excess of the secondary functional metabolites mentioned above but are not limited to phenolic compounds, terpenes, polyketides, and steroids due to their metabolism (Zhong and Xiao, 2009). There are various mushrooms whose secondary metabolites possess drug-like structures (i.e., Lipinski’s Rule of five compliant such as log P, number of hydrogen bond donors, molecular weight as well as the number of hydrogen bond acceptors) moreover might be considered as a major natural inspiration for drug discovery purposes (Leeson, 2012). Lipinski’s rule of five binding mechanisms works effectively using molecular docking studies. Mushrooms also represent a source of polysaccharides having immune-stimulating as well as anti-cancer properties. Globally, *Agaricus bisporus* is the most cultivated mushroom followed by *Flammulina velutipes, Lentinus edodes*, and *Pleurotus* sp. The production of mushrooms is continuously growing and globally China is the biggest producer (Chang and Miles, 2008; Aida et al., 2009; Patel and Goyal, 2012). Although, wild mushrooms are becoming extremely critical for their sensory and pharmacological characteristics (Ergomul et al., 2013).

In various parts of the world, mushrooms occur from arctic to tropic levels. There are some species that mainly occur in geographically restricted areas whereas others exist in widely geographically separated areas. Nevertheless, there are many species that seem to show a preference for a definite type of habitat. Mushrooms exist in swamps, or are primarily found in upland wooded areas and also in open areas like pasture, gardens, and lawns like *Pleurotus tuber-regium*, whereas others are found on wood (Lignicolous) like *Lentinus edodes*, on dung (Corpophilous) like *Coprinus lagopus*, and on dead leaves (Follicolous) or litter like *Tricholoma bayensis* and *Cortinarius melliolens* (Pant et al., 2021). There are also a few mushrooms that grow on the nasidiocarps of other mushrooms and are known as Fungicolus. Mushrooms are mainly found on wastes like composting materials, sawdust, and garbage (Gbolagade, 2006).

During the pandemic of SARs-CoV-2 the uptake of mushrooms has been increased because the regular intake of natural products such as *Grifola frondosa, Inonotus obliquus*, and *Lentinula edodes* with effective anti-inflammatory and antiviral peculiarities reduced the effect of SARs-CoV-2 (Negi et al., 2020; Shahzad et al., 2020; Sarangi et al., 2021).

Mushroom also contain various types of non-digestible carbohydrates such as raffinose, chitin, oligosaccharides, β-glucans, and also resistant starch (Manzi et al., 2001; Dikeman et al., 2005) but according to Samsudin and Abdullah, mushrooms have both digestible carbohydrates such as glucose, mannitol, glycogen, and trehalose as well as non-digestible carbohydrates such as mannans, β-glucans and chitin (Samsudin and Abdullah, 2019). Mineral composition of *Agaricus bisporus* and *Agaricus bitorquis* are identified by Saiqa et al. (2008) and also identified that these compositions are low in concentration of cobalt, chromium, lead, zinc, manganese, chromium, nickel, and copper whereas enriched with essential minerals including lithium, sodium, and potassium. Both of these compositions exhibit a high quality of carbohydrates, proteins, and lipids (Saiqa et al., 2008).

There are some mushrooms such as *Trametes versicolor*, *Schizophyllum commune*, *Flammulina velutipes*, *Ganoderma lucidum*, *Phellinus linteus, Lentinus edodes, Cordyceps sinensis, Inonotus obliquus*, and *Grifola frondosa* that possesses inhibitory effects toward cancer as well their immuneceuticals actions, mainly *via* the elevation of the immune system of the host. The activation of NK cells, macrophages, dendritic cells, T-cells, and the production of cytokines are involved in this process (Wasser, 2010). According to Hetland et al. (2011) *Agaricus blazei* is plentiful in the β-glucan and polysaccharides and has been disclosed to have antiallergic, anti-infection, asthmatic, and antitumor properties in mouse models, in inflammatory bowel disease patients, it cojoined with anti-inflammatory impact. These impacts are because of innate immune cells like dendritic cells, monocytes, enhancement of skewed Th1/Th2 balance, NK cells, and inflammation.

## Medicinal Mushroom in India

According to Rai et al. (2005) there are approximately 14,000 species known, out of which 2,000 species are safe for human consumption and 650 species have medicinal properties (Rai et al., 2005). In India and other developing countries that have a rich biodiversity of mushrooms, they are a boon for progress in the field of medicine, unemployment, and food due to various medicinal and nutraceutical mushrooms which are beneficial for the development of human health as minerals, drugs, food, and medicine (Rai et al., 2005; Sheena et al., 2005; Wani et al., 2010).

Currently, 35 species of mushrooms have been cultivated commercially whereas 20 species have been cultivated on an industrial scale. Mushroom diversity varies from typical *Agaricus* mushrooms with a stalk and umbrella-shaped top to the polypores, puffballs (Lycoperdon), Earthstars (Geastrum), and Stink Horns (Phalloides).

## Active Metabolite From Mushroom

### Polysaccharides

Mushroom polysaccharides are exploited and developed as a functional food substance such as ganoderan from *Ganoderma lucidum*, pleuran from *Pleurotus* species, lentinan from *Lentinus edodes*, calocyban from *Calocybe indica*, schizophyllan from *Schizophyllum commune* most of which are the representatives of D-glucans having common (1→3) or (1→6) β-linked glucose backbones (Villares et al., 2012; Badalyan, 2014). Commonly found monosaccharides in mushroom polysaccharides are mannitol, mannose, glucose, trehalose, rhamnose, xylose, galactose, arabinose, xylose, fucose, fructose, etc. (Valverde et al., 2015). Polysaccharides are one of the most common and potent compounds derived from mushrooms and exhibit various human-beneficial activities such as anti-inflammatory, antitumor, and immunomodulatory activities (Pant et al., 2020). The common functioning mechanisms of various mushroom polysaccharides that involves the activation of the above three activities are the activation of neutrophil, cytotoxic, cytokines (interferons, interleukins, and colony-stimulating factors), natural killer cells, monocytes, dendritic cells, and macrophages (Wasser, 2011). The health benefits of mushroom polysaccharides *via* the regulation of gastrointestinal and gut microbiota function (Prajapati et al., 2021). Mushroom polysaccharides as dietary fibers display an interaction process with colonized microbiota gut in gastrointestinal tracts that might change several variations of gut microbiota which impacted the health level of the host (Kong et al., 2016). Polysaccharides that obtained from the mushrooms might be degraded by the gut microbiota and some bacterial groups absorb them as an energy source that displays stimulation effects on the production of beneficial compounds such as short chains fatty acids like butyrate, valerate acid, acetate, and propionate and their propagation (Zhu et al., 2016; Ma et al., 2017). During the process of fermentation, the extracts of *Ganoderma lucidum* mostly composed of polysaccharides and have the ability to increase the abundance of *Bifidobacteria* (Yamin et al., 2012); Zhang et al. (2017) demonstrated that the Ganoderan is extracted from the *Ganoderma* sp. and having β-(1→3)-D-glucans with β-(1→6)-D-glucopyranosyl branches and the molecular weight is around 1.2 × 10^6^ Da to 4.4 × 10^6^ Da (Üstün et al., 2018; Ziaja-Sołtys et al., 2020). Additionally, due to the daily supplementation of *Ganoderma lucidum* polysaccharide strain, the abundance of bacteria like *Roseburia*, *Lachnospiraceae*, and *Lactobacillus* with health benefits to host enhanced significantly (Li et al., 2016). Colonized bacteria of the human colon utilized the β-glucans obtained from the mushroom and displays the potential to improve the health of humans by selectively changing the abundances of bacteria such as lactic acid bacteria and *bifidobacterial* (Wong et al., 2005). High molecular weight polysaccharides greater than 300 KDa that isolated from the extracts of water of *Ganoderma lucidum* mycelium which produce anti-obesity as well as microbiota-modulating effects like reduced endotoxin-bearing *proteobacteria* level as well as the ratio of *Firmicutes/Bacteroidetes*, decreased bodyweight, insulin resistance, and inflammation in mice fed a high-fat diet (Chang et al., 2015). The composition of the gut microbiota might be regulated by *Inonotus obliquus* polysaccharides as well as in mice having chronic pancreatitis (Hu et al., 2017).

Lentinan, from *Schizophyllum* sp. also has a β-(1→3)-glucan with a β-glucopyranosyl group linked by β-(1→6) linkage and molecular weight around 450 kDa (Pandya et al., 2019). Pleuran is a water- as well as alkali-soluble polysaccharide that is extracted from the *Pleurotus* sp. and has β-(1→3/1→6)-D-glucan or α-(1→3)-D-glucan structurally (Baeva et al., 2019). Grifolan extracted from the *Grifola* sp. of mushroom with gel-forming β-(1→3)-D-glucan. Molecular weight is around 770 kDa to 1650 kDa (Giavasis, 2014).

Kristiansen (2015) polysaccharide-K or PSK is extracted from the *Trametes* sp. with protein-bound β-glucan and having β-(1→4)-glucan with lateral β-(1→6)- glucopyranoside chains. It has a molecular weight of around 94 kDa (Zong et al., 2012). Another proteoglucan extracted from *Trametes* sp. is polysaccharide-protein complex. Polyporus polysaccharide is extracted from *Polyporus* sp. of mushroom with (1→3)- β-glucan backbone and (1→6)-β-glucopyranose side chain. Its molecular weight is around 1.6 × 10^5^ Da (Zong et al., 2012). Polysaccharides that extracted from the *Agaricus* sp. have several structural variants like an acidic β-(1→6)/α-(1→3)-glucan, an acidic β-(1→6)/α-(1→4)-glucan and β-(1→6)/β-(1→3)- glucan with very broad molecular weight starting from 380 kDa to 10,000 kDa (Giavasis, 2014).

It has been found that the polysaccharide extracted from edible mushrooms such as *Auricularia* (jelly ears fungi) has a β-(1→3)-D-glucan that is linked with two residues of β-(1→6)-D-glucosyl for every three main chain glucose moiety. It has a molecular weight of around 2.1 × 10^3^kDa (Miao et al., 2020). Polysaccharides that extracted from *Cordyceps* sp. having α-(1→4)- D-glucan linked with the branches of α-(1→6)-D-glucan with a molecular weight of around 1180 kDa (Zong et al., 2012).

### Proteins and Peptides

In mushrooms, proteins and peptides are the important bioactive nutraceuticals having various health benefits like some enzyme inhibition activities, increased in the absorption and digestion of exogenous nutritional ingredients and the immune function modulation to help the host defending the invasion of pathogens as well as the inhibition activities of few enzymes (Valverde et al., 2015). It has been found that the proteins and peptides in mushrooms have pharmaceutical potential such as ribonucleases, lectins, ribosome-inactivating proteins, laccases, and fungal immunomodulatory proteins (Xu et al., 2011). Lectins are the glycol or non-immune proteins that binds with the carbohydrates of cell surfaces and possess important activities like antiviral, antitumor, antifungal, immunomodulatory properties, antibacterial, etc. (Singh et al., 2014) whereas fungal immunomodulatory proteins are used as adjuvants for the treatment of tumors because of their important activity in suppressing the invasion as well as metastasis of tumor cells (Lin et al., 2010).

Ribosome inactivating proteins are able to inhibit the fungal proliferation as well as HIV-1 reverse transcriptase activity (Puri et al., 2012). Sanchez observed anti-tumor and immunomodulatory effects on breast cancer MCF7 cells, hepatoma HepG2 cells, human leukemic T cells (Sanchez, 2017). Further, there were also anti-viral studies observed on the reverse transcriptase activity on the immunodeficiency virus (HIV-1) which showed inhibitory function. Usually, edible mushrooms possess between 19 to 39% of protein by dry weight while being a part of a complex network of fungal cells (Reddy, 2016).

Dahima et al. (2020) demonstrated that the pleurostrin extracted from the *Pleurotus ostreatus* is a 7 kDa anti-fungal peptide. It has also been found that the cordymin extracted from the mushroom *Cordyceps militaris* and *Cordyceps sinensis* is an anti-inflammatory peptide having a molecular weight of around 10,906 kDa. (Elkhateeb and Daba, 2019; Ashraf et al., 2020). Xylose-specific lectins extracted from *Xylaria hypoxylon* with a molecular weight of around 28.8 kDa show anti-tumor as well as anti-mitogenic activities (Büttner et al., 2019). Laccases that are isolated from *Pleurotus ostreatus* and *Pleurotus eryngii* display an anti-viral property (Wang and Ng, 2006; Ilyicheva et al., 2020); Lynn (2017) has also been found that there are several FIPS that have been isolated successfully from different mushrooms like Fip-gts from *Ganoderma tsugae*, Fip-fve from *F. velutipes*, and Fip-vvo from *Volvariella volvacea*. According to Chang et al. (2010) and Zhao et al. (2020) Fip-fve has been applied successfully for tumor immunotherapy. A 9 kDa RIP Marmorin with anti-tumor properties has been isolated from the *Hypsizygus marmoreus* (Wong et al., 2018).

### Terpenes

Terpenes that are found from the mushrooms are regarded as volatile unsaturated hydrocarbon clusters that are categorized as mono, sesqui, di, and triterpenoids (Duru and Cayan, 2015). There are various types of sesquiterpenoids that are obtained from the mushrooms such as drimane, aristolane, sterpurane, cuparene, lactarane, spiro, bisabolane, fomannosane, and nordasinane. According to Duru and Cayan, mostly the diterpenoid mushrooms were detected as cyathane type while the triterpenoid compounds were detected as lanostane type that isolated from the mushrooms (Duru and Cayan, 2015). Various fungal sesquiterpenic molecules are effective against Leishmania mainly, *Eimeria tenella, L. donovani*, *L. infantum*, *Acanthamoeba castellanii*, *Trypanosoma brucei*, *T. cruzi*, *Neospora caninum*, *T. gondii*, and other parasites (Lenzi et al., 2018). *Lentinus* species can yield various sesquiterpenes. Lenzi et al. (2018) and Kumari et al. (2019) demonstrated that the sesquiterpenes panepoxydone and hypnophilin might be isolated from the ethyl acetate extracts of mushroom *Lentinus strigosus* whereas some of the sesquiterpenes such as dihydrohypnophilin, panepoxydione, and panepoxydone were isolated from the ethyl acetate extracts of fungus *Lentinus conatus*. According to Breitmaier (2006), Linalool is the monoterpene derivative with anti-bacterial activity. Sesquiterpenes are mainly composed of three isoprene units with general molecular formula C_15_H_24_. They are mainly produced by some fungi and plants. Geosmin is the common sesquiterpene produced by actinomycetes. Additionally, these mushroom terpenes have been associated with various health benefits such as antiviral (Asakawa et al., 2014), anticholinesterase (Dundar et al., 2015), anticancer (Song et al., 2013; Klaus et al., 2017), antioxidant (Boonsong et al., 2016; Kumari et al., 2021), antimalaria (Ozturk et al., 2015), anti-inflammatory (E1 Enshasy and Hatti-Kaul, 2013; Elsayed et al., 2014) activities. These multiple sesqui, di, and triterpenoids were obtained from various species of mushrooms like *Ganoderma* species, *F. velutipes* and *Pleurotus*. There are two main terpenes such as Flammulinolides and Flammulinol that are isolated from *F. velutipes* having cytotoxicity against the three tumor cell lines KB, HeLa, and HepG2 while terpenes that isolated from the *Pleurotus* species revealed very essential bioactivities like two sesquiterpenoids as well as five monoterpenoids having anti-inflammatory effects.

Lee et al. (2011) found that n-butyl ganoderate H as well as methyl ganoderate A acetonide from *G. lucidum* owes the ability of anti-acetylcholinesterase effect for the Alzheimer’s and related neurodegenerative diseases.

### Phenolic Compounds

The phenolic compounds of mushrooms which are regarded as aromatic hydroxylated compounds with one or more hydroxyl groups as well as aromatic rings mainly comprise tannins, phenolic acids, oxidized polyphenols, hydroxybenzoic acids, lignans, flavonoids, stilbenes, and hydroxycinnamic acids (D’Archivio et al., 2010).

Additionally, these compounds provide protection against various degenerative disorders like cardiovascular diseases, cancer, brain dysfunction, and aging (Finimundy et al., 2013). They also have an excellent antioxidant capacity as well as antiviral, anti-inflammatory, antiatherogenic, cardioprotective, anticancer, antimicrobial, antithrombotic, antiallergenic and vasodilator effects (Balasundram et al., 2006; Ferreira et al., 2009; Heleno et al., 2012a). From the edible mushroom such as *Phellinus baumii Pilat* belongs to Hymenochaetaceae family, three phenolic compounds have been found that have the ability to inhibit LPS-stimulated nitric oxide (NO) production in RAW264.7 cells (Lee et al., 2017).

From the mushroom *Phellinus* species, the phenolic compound Hispidin has been derived which possesses anti-inflammatory activities *via* suppressing the ROS mediated NF-κB pathway in the macrophage cells of mouse (Shao et al., 2015); Chang et al. (2011) isolated phenolic compounds from the mushroom *Phellinus linteus* showed the anti-inflammatory mechanisms of decreasing the level of MDA in the edemapaw by enhancing the effect of SOD, GPx, and GRx in the liver, further the suppression of TNF-α and NO. Next, Patel and Goyal (2012) extracted hispolon from *Phellinus* species which induce epidermoid and gastric cancer cell apoptosis, and regardless of p53 status, hispolon inhibited bladder cancer and breast cell growth. According to Lai et al. (2010) the dichloromethane extracted from *Ganoderma lucidum* composed of phenolics, flavonoids, alkaloids, and terpenoids and also displayed anti-human papillomavirus 16 (HPV 16) E6 oncoprotein effect. When epidermoid cervical carcinoma (CaSki) cells are treated with the crude dichloromethane extracts, HPV 16 E6 production gets suppressed. In eight types of edible mushrooms such as *Pleurotus ostreatus*, *Cantharellus cibarius, Agaricus bisporus*, *Lactarius deliciosus, Boletus edulis*, *Craterellus cornucopioides*, *Hygrophorus marzuolus*, and *Calocybe gambosa* the contents of total flavonoid and total phenolic has been evaluated.

### Polyunsaturated Fatty Acids

Ergosterol is one of the major sterols produced by mushrooms and has shown important antioxidant properties (Guillamon et al., 2010). It also plays an important role in the prevention of cardiovascular diseases (Kalac, 2013). They are detected as tocopherols and considered as effective as well as novel natural antioxidants and have major biological activities for the protection against microbial and cardiovascular diseases and degenerative malfunctions (Heleno et al., 2012a, 2015b; Jaworska et al., 2015). Linoleic acid participates in various physiological functions such as decreasing the inflammatory level *via* inhibiting the production of NO and also suppressing the expression of pro-inflammatory cytokines like TNF-α, IL-6, IL-1β, and NOS2 in RAW 264.7 cells (Saiki et al., 2017). The decreasing impact on the Alzheimer’s disease risk correlates with its inhibition effects against the acetylcholinesterase (AChE) and butyrylcholinesterase (BChE) (Ozturk et al., 2014).

### Carbohydrates

Xylose, maltose, rhamnose, trehalose, mannitol, arabinose, fructose, sucrose, fucose, glucose, and mannose are the properties of carbohydrates that have been quantified in various mushrooms (Zaidman et al., 2005; Zhang et al., 2007; Ferreira et al., 2009; Heleno et al., 2012b). The antitumor polysaccharides which are isolated from mushrooms can be acidic or neutral in nature and have strong antitumor action. Moreover, they also differ in their chemical structures. Glycans which extend from homopolymers to highly complex heteropolymers and exhibit the antitumoral effect. Due to the activation of the immune system, mushroom polysaccharides have antitumor action. Wasser (2002) and Zhang et al. (2007) observed that mushroom polysaccharides cannot kill the tumor cells directly. These compounds prevent the body from stress, moreover, they can produce half the reduction in tumor size and extend the survival time of tumor-bearing mice.

According to De Groot and Hellingwerf (2001), McIntosh et al. (2005), Schmid et al. (2001)β-glucans are the main polysaccharides that are found in mushrooms whereas 50% of the fungal cell wall mass is founded by β-glucans. For the industry, it is very essential due to their excretion into the cell growth medium and making their recovery. Its chemical characterization and purification are very simple. These β- glucans are highly responsible for the antioxidant, anticholesterolemic, anticancer, neuroprotective, immunomodulating activities of several edible mushrooms (Falch et al., 2000; Ishibashi et al., 2001; Kataoka et al., 2002; Khan et al., 2013). They are also essential in protection from cancer as well as infectious diseases and also recovered the aid patients from radiotherapy and chemotherapy (Manzi et al., 2001; Chen and Seviour, 2007). Consequently, reinforcing as well as activating the host immune system is the best strategy for inhibiting the cancer cells’ growth (Daba and Ezeronye, 2003; Finimundy et al., 2013).

### Lipids

Mostly the edible mushrooms contained polyunsaturated fatty acids and help to decrease the serum cholesterol level. Tocopherols are the natural antioxidant compounds found in the lipidic fraction as they act as free radical scavenging peroxyl components produced from several reactions. These antioxidants are protective against cardiovascular diseases, degenerative malfunctions, and cancer. Linoleic acid, an essential fatty acid to humans, decreases triglyceride levels, arthritis, cardiovascular diseases, and blood pressure (Hensley et al., 2004; Ferreira et al., 2009; Heleno et al., 2012a; Reis et al., 2012).

## Unknown Unidentified Metabolite

Many researchers have identified some unknown metabolites such as unknown (1), unknown (2), unknown (3), unknown (4), unknown (5), unknown (6), unknown (7), unknown (8), unknown (9), unknown (10), unknown (11), unknown (12), unknown (13), unknown (14), unknown (15), unknown (16) (Satria et al., 2019) and also some other metabolites such as ganoderenic acid A, B, C, D, F, K; ganoderic acid A, B, C2, H, I, K; 12-acetoxy-7-hydroxy-3,11, 15-trioxolanost-8,16,24-trien-26-oic acid; 12-acetoxy-3-hydroxy-7,11,15-trioxolanost-8,16,24-trien-26-oic acid; 3,12,20- trihydroxy-7,11,15-trioxolanost-8,16,24-trien-26-oic acid; 12- hydroxy-3,7,11,15,23-pentaoxo-lanost-8-en-26-oic acid; 3,12- dihydroxy-4,4,14-trimethyl-7,11,15-trioxolanost-8,9,20,22-en- 26-oic acid; 12-acetoxy-7-hydroxy-3,11, 15-trioxolanost-8, 20-dien-26-oic acid; ganolucidic acid A; 3,7-dihydroxy-11,15, 23-trioxolanost-8,16-dien-26-oic acid; 7,12-dihydroxy-3,11,15, 23-pentaoxo-lanost-8,20-dien-26-oic acid; 12-acetoxy-3-hydroxy-7,11,15,23-tetraoxolanost-8,20-dien-26-oic acid and 12-acetoxy-3,7,11,15,23-pentaoxo-lanost-8,20 -dien-26-oic acid (Satria et al., 2019). There are various primary and secondary metabolites that are present in mushrooms such as proteins, lipids, carbohydrates, polyunsaturated fatty acids, alkaloids, terpenes, triterpene, sesquiterpenes, flavonoid, flavanone, saponins, alkaloids, polysaccharides, anthraquinone, tannins, steroids, glycoproteins, polyketides, phenolic compounds, melanin, β-glucan, dietary fibers, lectins, and many other compounds. Some of them are mentioned above but still, there are several other unknown metabolites present in the mushroom which do not report yet. There remains a need to find out other important bioactive components that can be essential/beneficial for human health.

## Small Molecules

Valko et al. (2007) found that mushrooms have small molecules such as organic germanium, catechols, quinones, steroids, selenium, amines, sesquiterpenes, cerebrosides, and triacylglycerols. Lindequist et al. (2005b) demonstrated that the *Hericium* sp. have various small molecules that include compounds such as aldehyde derivative of 4-chloro-3,5-dimethoxybenzaldehyde and an erinacerin V alkaloid having a molar mass of 257 g/mol as well as an aldehyde derivative of 4-hydroxychroman having a molar mass of 206 g/mol. 5- hydroxy-6-(1-hydroxyethyl)isobenzofuran-1 (3H)-one, erinacine, 2-chloro-1,3-dimethoxy-5-methylbenzene, 4-chloro-3,5-dimethoxybenzoic acid (4-chloro-3,5-dimethoxyphenyl) methanol, Molecules 2021, 26, 251 18 of 24 3, 6-bis(hydroxymethyl)-2-methyl-4H-pyran-4-one, etc.

Stamets (2000a) and Zhang et al. (2016) demonstrated that the North American mushrooms have several other small molecules such as hexadecanoic acid, orenalline from *Cortinarius armillatus*, pentadecanoic acid, octadecanoic acid from *Pleurotus djamor*, 2E, 4E- octadecadienoic acid. Except for the orenalline, there are various compounds that have not been assessed for toxicity as well as bioactivity. Nephrotoxin is considered to evaluate the toxicity of orenalline (Gadgil, 2005); Lallawmsanga et al. (2016) has been found that the coral mushroom *Ramaria cystidiophora* (Southwestern British Columbia, Canada) has four new compounds that belong to butanolide groups called ramariolides A-D. It has been found that Ramariolides A has antimicrobial activities against *Mycobacterium tuberculosis* as well as *Mycobacterium smegmatis* (Lallawmsanga et al., 2016).

Deng et al. (2009) found that syringic acid and syringaldehyde are the two molecules having a molar mass of 198 and 182 g/mol correspondingly from the ethanol extract of *E. Granulates* fruiting bodies. Both the compounds have possible toxicity that evaluated on macrophage HL-60 cells and Raw 264.7 cells. Deng et al. (2009) demonstrated that both syringic acid as well as syringaldehyde has no effect on HL-60 as well as on Raw 264.7 cells viability of up to 31.25 and 25 μg/mL correspondingly. According to Mothana et al. (2003) and Maia et al. (2015) found that Confluentin (326 g/mol), grifolin (328 g/mol), and neogrifolin (328 g/mol) have antibacterial as well as growth-inhibitory activities when isolated from the extracted ethanol of the fruiting bodies of *A. Flettii*. Whereas *J. hirtus* have A lanostane-type triterpene 3, 11 – dioxdanosta- 8,24(Z)-diene-26-oic acid having molar mass (468 g/mol) and this triterpene inhibits the growth of gram-positive bacteria such as *Enterococcus faecalis* as well as *Bacillus cereus* (Maia et al., 2015).

Overholts (1939) demonstrated that the fungus *U. criterium* that is found in the North American cup has three new bisnaphthalene subclass compounds known as urnucratins A-C, and that this urnucratin A is active against the *Streptococcus pyogenes, methicillin-resistant Staphylococcus aureus*, and *vancomycin-resistant Enterococcus faecium.* Masuda et al. (2008) found that a fungus *F. pinicola* in North America has a new lanostane triterpenoid such as 25-dien-21-oic acid as well as 3-oxo-24-methyl-5α-lanost-8 with activity against the *Bacillus cereus*. In this study, Masuda et al. (2008) also demonstrate one known ergostane steroid and four known lanostane triterpenoids having anti-bacterial activities.

## Medicinal Mushroom Identification

Morphologically mushrooms can be characterized/identified on the basis of their size, color (white, black, yellow, brown, cream, pink and purple-brown, etc.), consistency of the stalk and cap, chemical tests or reactions, spore color in mass, mode of attachment of the gills to the stalk (Carluccio, 2003; Fuhrer, 2005). Mushrooms can also be identified on the molecular level by Internal Transcribed Spacer (ITS) regions of gene sequencing. This ITS region is one of the most frequently used molecular markers for species delimitation in fungi. This ITS region is used to identify and compare the fungi that are present within the sample from genus to species level as it has a conserved sequence (Carlson et al., 2014).

There are various other techniques that are used for the identifications of mushroom such as microscopy, thin-layer chromatography, nuclear magnetic resonance spectroscopy, DNA sequencing, high-pressure liquid chromatography, chemical fingerprinting, ultra-performance liquid chromatography, high-performance thin-layer chromatography, liquid chromatography quadrupole time-of-flight mass spectrometry, gas chromatography-mass spectrometry, and fourier transform infrared spectroscopy (Toh Choon et al., 2012; Awadasseid et al., 2014; Hennicke et al., 2016).

**FIGURE 7:**
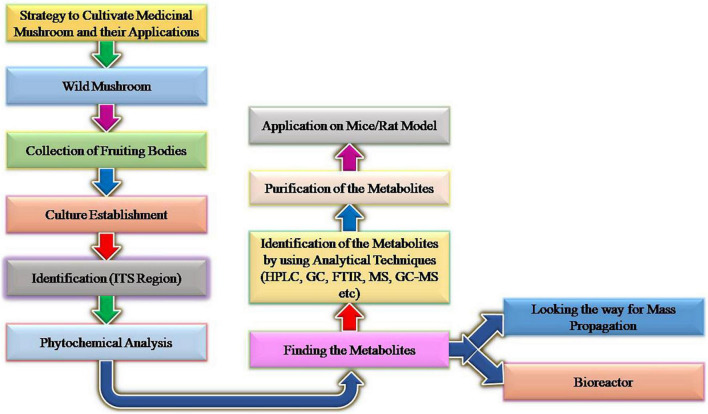
Representing identification and phytochemical analysis of medicinal mushroom.

Recently matrix-assisted laser desorption ionization-time of flight mass spectrometry (MALDI-TOF MS) technique has been developed rapidly which is widely used in clinical laboratories for the identification of fungus. This MALDI-TOF MS technique has been possessed as a promising tool for the identification of fungus because of its rapid performance, cost-effectiveness, and high throughput (Cassagne et al., 2016). Represents the identification and phytochemical analysis in Figure 7.

## Potential Medicinal Mushrooms

### Coriolus versicolor

*Coriolus versicolor* is one of the dried fruiting body or mycelia of *Coriolus versicolor.* This *Coriolus versicolor* is visible as a fan-shaped mushroom having a wavy margin and colored concentric zones. This obligate aerobe is commonly found year-round on dead logs, branches, stumps, and tree trunks. This fungus occurs all over the wooded temperate zones of North America, Asia, and Europe and is the most common shelf fungus in the Northern Hemisphere. This mushroom belongs to the family Basidiomycotina. This *Coriolus versicolor* has many different names such as *Boletus versicolor, Polystictus versicolor, Trametes versicolor, Agaricus versicolor, Poria versicolor, Yun-zhi* (China), i.e., cloud-like mushroom probably due to its wavy surface that covered with fluff, *Kawaratake* (Japan) which means mushroom by the riverbank, *Turkey-tail* (North-America) as it fan-shaped zoned having shades of white, gray and brown in color on the upper surface and on dead logs, it grows in overlapping clusters and *Polyporus versicolor*.

This *Coriolus versicolor* fruiting body has brackets 3 to 5 cm across which are flattened, tough, thin, and semi-circular whereas young brackets are flexible. Mostly these brackets occur in tiers and spread along the branches. The upper surface of the *Coriolus versicolor* fruiting body is velvety and attractively marked having concentric zones of several colors, i.e., black, gray, yellow, greenish, and brown. Mostly the margin of the fruiting body is wavy. The mushroom has white spores which are cylindrical (4–6 μm × 2–2.5 μm) and oblong. The spores and fruiting bodies do not form in agitated submerged culture and grow as pelleted or dispersed mycelium. More than 120 strains of *Coriolus versicolor* have been recorded (Soothill and Fairhurst, 1977). The fruiting body of *Coriolus versicolor* is harvested for its medicinal and nutritional values just like all other mushrooms. This fungus is known to have potential pharmacologically active secondary metabolites which belong to small molecular weight compounds in addition to the minerals and major macromolecules, i.e., carbohydrates, lipids, and proteins (Habtemariam, 2020). In European origin, the phenolic composition of *Coriolus versicolor* fruiting body about thirty-eight phenolic compounds have been identified by HPLC-MS/MS- based study belonging to the hydroxycinnamic acids and flavonoid such as flavonols, bioflavonoids, isoflavonoids, flavones (Janjusevic et al., 2017).

The most common species within the genus is *Trametes versicolor* which is commonly known as Turkey tail and has been reported on 295 woody plant species including angiosperms and conifers (Grand and Vernia, 2002). *Trametes versicolor* belongs to the domain ‘eukaryota’ and kingdom ‘fungi’ and has phylum and subphylum, i.e., Basidiomycota and Agaricomycotina whereas belonging to the class Agaricomycetes and subclass Agaricomycetidae. It has order polyporales and belongs to the family polyporaceae and species *Trametes versicolor*. This *Trametes versicolor* is commonly widespread across Ireland and Britain and this Turkey tail fungus occurs all over the mainland of Europe from northern Scandinavia right down to the Mediterranean region., This wood-rooting fungus is also found in Asia and North America.

**FIGURE 8:**
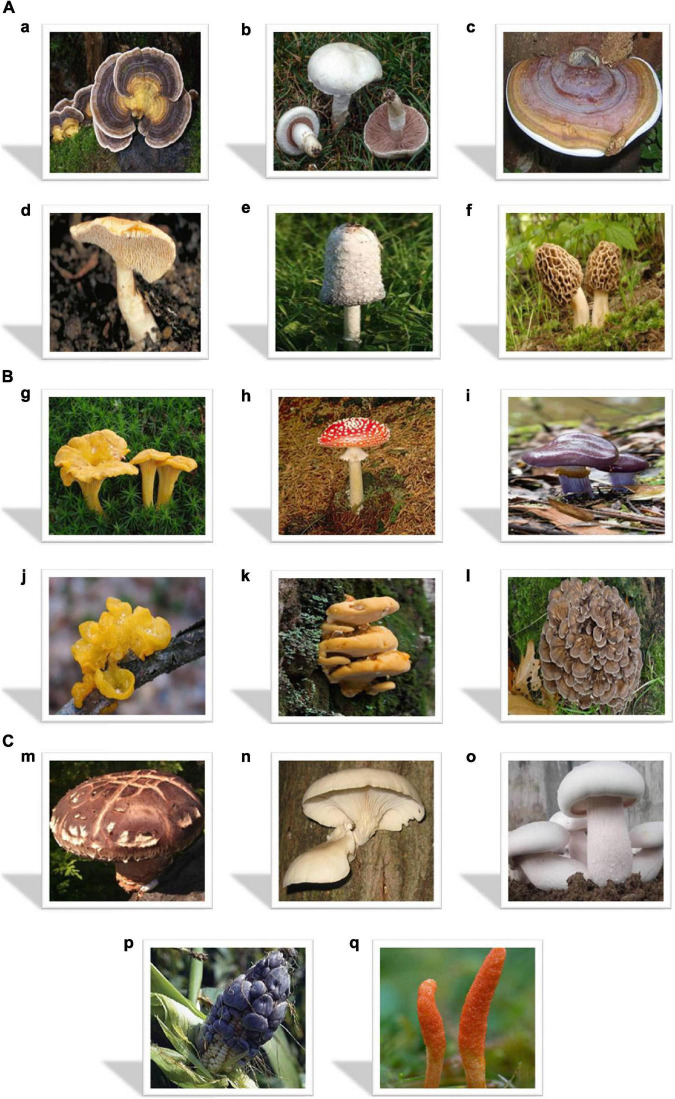
**(A)** Representation of Medicinal Mushrooms **(a)** *Coriolus versicolor* (Paul et al., 2008) **(b)** *Agaricus* sp. (Glamočlija et al., 2015) **(c)** *Ganoderma* sp. (Hong and Jung, 2004) **(d)** *Hydnum* sp. (Paul et al., 2008) **(e)** *Coprinus* sp. (Coprinus Pers, 2015) **(f)** *Morchella* sp. (Raman et al., 2018). **(B)** Representation of Medicinal Mushrooms **(g)** *Cantharellus* sp. (Pilz et al., 2003) **(h)** *Amanita* sp. (Tulloss, 2015) **(i)** *Cortinarium* sp. (*Cortinarius* (Pers.) Gray, 2020) **(j)** *Tremella* sp. (Rea, 1922) **(k)** *Rigidoporus* sp. (Rigidoporus, 1905) **(l)** *Grifola* sp. (Cetto, 2008). **(C)** Representation of Medicinal Mushrooms **(m)** *Lentinus* sp. (Wells, 2008) **(n)** *Pleurotus* sp. (Chang and Miles, 2004) **(o)** *Calocybe* sp. (Kirk et al., 2008) **(p)** *Huitlacoche* sp. (Spraker, 2013) **(q)** *Cordyceps* sp. (Pat O’Reilly, 2016).

In 1753, Carl Linnaeus originally described *Trametes versicolor* and gave it the binomial name *Boletus versicolor* and further, it was as recently as 1939 that this species renamed as *Trametes versicolor* by Czech mycologist Singer (1975). *Trametes versicolor* is one of the cosmopolitan genera of white-rot polypores and the most familiar genera of polypores, its species-level taxonomy is unsettled. These species are available in about all forest ecosystems and are also found often on many genera of hardwoods in every part of northern temperate forests. Apart from their noteworthy role in the ecosystem’s recyclers and decomposers, the mushrooms have various biological activities including antibacterial, anticancer, insecticidal, antioxidant, anti-proliferative, anticoagulant, antifungal, antidiabetic, hepatoprotective, antiparasitic, antiviral, and anti-inflammatory properties (Atomachi, 1988). More than one hundred and twenty strains of *Coriolus versicolor* have been recorded in the Compendium of Chinese Materia Medica. This *Coriolus versicolor* has cold properties and is slightly sweet in taste, exerting its effects through the spleen and liver according to the theory of traditional Chinese medicine practices (Wasser, 2010). *Coriolus versicolor* is also useful for strengthening the physique, removing toxins, dispelling heat, increasing the host’s immune function, and enhancing the energy and spirit. *Coriolus versicolor is* often used for several types of cancers, upper respiratory, digestive, and urinary tract infections and chronic hepatitis in the clinical practices of traditional Chinese medicine. Picture of *Coriolus versicolor* shown in Figure 8Aa.

### *Agaricus* sp.

*Agaricus campestris* is frequently known as meadow or field mushroom. *Agaricus campestris* has pileus 3 to 6 cm wide. This *Agaricus campestris* is hemispherical to convex in nature and becomes planar at maturity. *Agaricus campestris* when young are pure white in color and become yellowish at the center with age and sometimes depressed at the center. Its surface is smooth, dry, and silky fibrillose whereas sometimes it is a few grayish to brown fibrils or fibrillose over the cap surface. Its complete margin is expanded beyond the gills frequently hung with veil remnants. On exposure, *Agaricus campestris* has firm, thick, reddening, and white flesh. It has a mild taste and a fungal odor. It has close Lamellae and it is pinkish white in button then bright pink and then becomes chocolate brown. The length of its stipe is 3–7 cm long and 0.5–1 cm diameter. It has been found that at base it is attenuated or bulbous and centric. It is white to whitish in color and has a smooth surface above the veil. It often has few fibrils below which are stuffed or hollow, silky, and the context is white slight reddening on exposure. At its best it has an annulus of white color which has a thin and skirt-like appearance. The print of its spore is chocolate brown. The spore is 5.5–7 μm × 3.5–5 μm and elliptical.

*Agaricus* mushroom is a fungus that is used for ongoing liver disease, cancer, digestive problems, high cholesterol, type 2 diabetes, arteriosclerosis, bloodstream disorders, heart disease, osteoporosis, and stomach ulcers. This mushroom is also used to boost up the immune system as well as for physical and emotional stress. In *Agaricus campestris*, the presence of insulin-releasing, anti-hyperglycemic as well as insulin-like activities has been reported. It also has been found that the lectins that isolated from the *Agaricus campestris* enhance the insulin that releases by isolated ret islets of Langerhans (Ewart et al., 1975; Ahmad et al., 1984a,b). It is also used in the treatment of diabetes (Gray and Flatt, 1998; Usman et al., 2021). Picture of *Agaricus* sp. shown in Figure 8Ab.

### *Ganoderma* sp.

*Ganoderma* species is one of the facultative parasites that live as saprobe by roots of trees, feeding off as well as rooting stumps. They are pathogenic and wood-decaying fungus that causes stem and root rot in the perennial crops as well as butt root (Coetzee et al., 2015). The species of *Ganoderma* also plays a vital role in the breakdown of woody plants for the mobilization of nutrients. It is also useful in bioremediation due to the lignocellulose decomposing enzyme’s mechanism and also useful in bioenergy production (Coetzee et al., 2015; Bijalwan et al., 2020).

The fruiting body of *Ganoderma* contains polysaccharides that are useful in the inhibition of tumor growth. One of the major constituents is the glucans present in the cell wall of fungi. *Ganoderma* enhances the bodily resistance against tumor growth and also the immunity function. It also induces interferon production as well as kills the cells of the tumor within the body (Jong and Birmingham, 1992). It is also used in the treatment and prevention of diabetes, infections, cancer, immune system disorders, hepatoprotection and bacterostasis, etc. (Deepalakshmi and Mirunalini, 2011). Studies have shown that the regular consumption of *Ganoderma* has been linked to prolonged life, relaxed body, and alleviation of the symptoms of aging (Yuan et al., 2018).

*Ganoderma lucidum* have a 3 to 23 cm wide pileus. It is kidney-shaped and elongated. At maturity, it is more or less fan-shaped and becomes red to reddish-brown when gets mature. When it is young, it often has zones of bright yellow and white toward the margin. It has up to 2 cm deep tubes as well as 0.1 cm of pores. It is whitish in color, usually bruising brown. It has 3 to 12 long stipes 1 to 2 cm thick. It is cylindrical, dark red to black with varnished crust, has a brown print spore, smooth and twisted in nature (Vishwakarma et al., 2011).

*Ganoderma lucidum* is widely used in the treatment of bronchitis, gastric ulcer, hepatopathy, asthma, insomnia, chronic hepatitis, nephritis, arthritis and hypertension, weakness, fatigue, and cough (Halpern and Miller, 2002). It is also used in India in the treatment of joint pain (Harsh et al., 1993). It also has some very significant properties like hepatoprotective, anti-cancer, anti-atherosclerosis, very strong immunomodulating effects, anti-oxidant, anti-HIV, nephroprotective, anti-tumor, anti-hepatotoxic, cardiovascular, and respiratory properties (Chang, 1995; Stamets, 2000b). It is also found out that the substances extracted from the mushrooms can decrease the level of blood pressure, blood sugar level, inhibition of platelet aggregation as well as blood cholesterol, etc. (Ajith and Janardhanan, 2007). A picture of *Ganoderma* is shown in Figure 8Ac.

### *Hydnum* sp.

*Hydnum* is the genus of fungi that belongs to the Hydnaceae family and are significant for their unusual spore-bearing structures of teeth other than gills. *Hydnum repandum* and *Hydnum rufescens* are the two best-known edible mushrooms. *Hydnum repandum* has a pileus of 3 to 10 cm in diameter. It is convex and becomes nearly a plane having a central depression. Its surface is smooth to slightly scaly, dry, cream to buff-orange, or planer. In the beginning, its margin is inrolled and at maturity it becomes lobed to undulating and bruising to orange-brown. *Hydnum repandum* is fleshy white and often discolors to yellowish when it is bruised or exposed. Its taste and odor are peppery or mild (Vishwakarma et al., 2011).

Commonly, *Hydnum repandum* is known as hedgehog mushroom or wood hedgehog. Repandiol compound extracted from the *Hydnum repandum* shows cytotoxic activity against a variety of tumor cell types, mainly colon adenocarcinoma cells (Takahashi et al., 1992). Chloroform extracted from this mushroom has some mild antibiotic activities against *Staphylococcus aureus*, *Bacillus subtilis, Enterobacter aerogenes*, and *Staphylococcus epidermidis* while extract of ethanol has mild activity only against the *Bacillus subtilis.* Picture of *Hydnum is* shown in Figure 8Ad.

### *Coprinus* sp.

*Coprinus* is one of the tiny mushroom genera which forms fungi that consists of *Coprinus comatus*. This *Coprinus comatus* is 4 to 10 cm broad, oval, and cylindrical in nature and elongated as conical or bell-shaped as well as becomes revolute or torn at the margin. Its surface turns white to pinkish toward the margin and then becomes blackish. It has a smooth center as well as ochraceous cream, pale or buff fulvous at first, and has shaggy white to pale brownish scales often darkest at the tips. It has decurved margins that inroll at maturity. It has no or slightly pleasant taste with a mild odor (Vishwakarma et al., 2011).

Commonly, this fungus is known as shaggy ink cap, shaggy mane, or lawyer’s wig. This fungus has various bioactive functions such as its consumption helps to regulate the blood glucose level, hypoglycemic and has antitumor, antioxidative, hypolipidemic, antibacterial as well as immunomodulation effects (Bailey et al., 1984; Fan et al., 2006; Wei and Van Griensven, 2008). Picture of *Coprinus* is shown in Figure 8Ae.

### *Morchella* sp.

The genus *Morchella* belongs to the family Morchellaceae. They have about 80 various species; one of them is *Morchella esculenta* that has a 3 to 5 cm diameter of pileus and is 4 to 8 cm long. This mushroom species is subglobose to ovoid and pale brownish cream and pale brown to grayish brown in color. Its surface is covered with irregularly interwoven pits of several colors or rounded pits. Its edge is thick, rounded and yellow or white in color. Its stipe is 3 to 6 cm long, having a 2 to 4 cm diameter. At base, it is bulbous, centric, or attenuated. Firstly, it appears as white in color then with age it becomes ochraceous. Just beneath the pileus, it has wrinkled as well as grooved scurfy tufts as well as it is also brittle and hollow. It has white and thin flesh with a yellow spore print. Spores are 11.5–14 μm × 19.5–23 μm ellipsoid (Vishwakarma et al., 2011).

*Morchella esculenta* is one of the edible morel mushrooms commonly known as Guchhi. This mushroom is used in food, medicines, and also health care. *Morchella esculenta* shows anti-inflammatory as well as antitumor activity against both ascites as well as solid tumors of ethanolic extracts whereas methanolic extracts prepared from its mycelia show a high content of total phenols as well as high antioxidant activity (Mau et al., 2004; Nitha et al., 2007). A picture of *Morchella* is shown in Figure 8Af.

### *Cantharellus* sp.

*Cantharellus* is the genus of edible mushrooms that are locally known as golden Chanterelle or Chanterelle, which refers to the species *Cantharellus cibarius*. This *Cantharellus* mushroom belongs to the family Cantharellaceae.

The species *Cantharellus cibarius* has a pileus of 3 to 10 cm in diameter. At first, it appears as convex with an inrolled margin and then often becomes funnel-shaped along with wavy-margins. It is pale yellow to egg-yolk yellow to almost orange in color having some small appressed fibers. It is mildly peppery in taste as well as having a fruity odor. Underneath its smooth cap on the lower surface, it has gill-like ridges which run almost all the way down its stipe that tapers down seamlessly from the cap. It has a 3 to 5 cm long stipe 1 to 2 cm thick in diameter. It is thin, solid, and smooth concolorous the pileus. It has pale yellow to creamy white spore prints. Spores are 7–10 μm × 4–6 μm smooth and ellipsoid (Vishwakarma et al., 2011). This species of mushroom has excellent antihyperglycemic, antioxidant, wound healing, antimicrobial, iron-chelation, cytotoxicity, anti-hypoxic, anti-inflammatory activities (Kumari et al., 2011). Picture of *Cantharellus* shown in Figure 8Bg.

### *Amanita* sp.

*Amanita* genus has around 600 species of agarics and it belongs to the family Amanitaceae. The edible species of mushrooms are *Amanita rubescens, Amanita fulva, Amanita calyptrate, Amanita jacksonii, Amanita vaginata, Amanita caesarea* whereas inedible mushroom species are *Amanita citrina, Amanita volvata, Amanita atkinsoniana, Amanita albocreata, Amanita excelsa, Amanita sinicoflava, Amanita longipes, Amanita onusta, Amanita franchetii, Amanita flavorubescens, Amanita rhopalopus, Amanita silvicola, Amanita spreta*, etc. (Phillips, 2010). There are some poisonous species, some lethally so, of *Amanita* such as *Amanita farinose, Amanita brunnescens, Amanita pantherina, Amanita cokeri, Amanita ceciliae, Amanita crenulata, Amanita muscaria, Amanita porphyria, Amanita frostiana, etc., Amanita ocreata, Amanita smithiana, Amanita abrupta, Amanita subjunguillea, Amanita virosa, Amanita proxima, Amanita exitialis, Amanita arocheae, Amanita magnivelaris, Amanita bisporigera, Amanita phalloides, Amanita smithiana, and Amanita verna* respectively (Zeitlmayr, 1976; Surayot et al., 2021).

The species *Amanita vaginata* has a pileus 9–10 cm × 3–5 cm in size and brown in color. Their cap shapes are usually convex and have smooth as well as round edges. It has been found that its cap has a fleshy brown color. Hymenophores are not present on the underside. In this mushroom, regular-shaped white colored gills present. It has a brown color stipe with 5 to 7 cm and 2 to 3 cm length and width correspondingly. Light brown color, single welled, round to oval shaped spore present having 7–8 μm × 5–6 μm size (Meena et al., 2020).

*Amanita* species have various biological activities like antitumor, pesticidal, cytotoxic, antioxidant, anticancer, antibacterial, acetylcholinesterase, esterolytic, antiviral, activity, anti-larvicidal, antifungal, anti-inflammatory properties (Sevindik et al., 2019). Picture of *Amanita* shown in Figure 8Bh.

### *Cortinarius* sp.

*Cortinarius* is one of the worldwide distributed genera of mushrooms that belongs to the family Cortinariaceae (*Cortinarius* (Pers.) Gray, 2020). It is supposed to be the major genus of agarics that contains over 2,000 widespread species (Kirk et al., 2008). The species *Cortinarius corrugates* having a pileus 22–23 cm × 8–9 cm in size and ash colored. This mushroom is ovate in shape with a grooved edge. There are hymenophores beneath the cap whereas on the underside of the cap regular-shaped gills are present. The stipe of the mushroom appears as milky white, on the upper part of the stipe an anal or black ring is present while on the lower part the volva is absent (Meena et al., 2020). Picture of *Cortinarius* shown in Figure 8Bi.

### *Tremella* sp.

The mushroom genus *Tremella* belongs to the family Tremellaceae. All species of *Tremella* are the parasites of other fungi and mostly produce as anamorphic yeast states when producing Basidiocarps. *Tremella* is colloquially classed among the gelainous or jelly fungi. *Tremella fuciformis* and *Tremella aurantialba* are the two species that are commercially cultivated for food (Zhang et al., 2009). *Tremella fuciformis is* locally known as silver ear fungus or snow fungus. It is tasteless in nature and has a gelatinous texture (Shu-Ting and Philip, 2004). *Tremella fuciformis* may have some medicinal important activities like fighting cancer, combating obesity, anti-aging, lowering cholesterol, protecting nerves, and acting as an anti-inflammatory. Picture of *Tremella* shown in Figure 8Bj.

### *Rigidoporus* sp.

*Rigidoporus* is a genus of the fungi that belong to the family Meripilaceae. This fungi forms flattened mycelia of white color which is 1 to 2 mm thick and grows on as well as adheres to the surface of root bark. The species *Rigidoporus microporus* is 20 cm wide, faintly velvety, leathery in nature. It has a broadly attached shelf as well as imbricate with the substrate. Its color changes from orange to brown or red and later becomes faded (Liyanage, 1997). In the absence of any woody substrates, these *rigidoporus* grow fast and spread several meters *via* the soil (Oghenekaro et al., 2014). Rigidoporous have several biological properties like mitogenic activity, anti-hepatitis B surface antigen effect, plasma clotting activity, activation of alternative pathway complement, and tumor suppressive effects (Chang et al., 2011). Picture of *Rigidoporus* shown in Figure 8Bk.

### *Grifola* sp.

The genus *Grifola* belongs to the family Meripilaceae. The species *Grifola frondosa* is locally called hen of the woods. This mushroom grows in clusters with a grayish to brown cap which appears as curled or spoon-shaped along with wavy margins of 2 to 7 cm wide at the base of the trees, especially oaks. It is 60 cm in size. Each cap bears 1 to 3 pores per mm at the under-surface with the tubes which are rarely deeper than 3 mm. Milky white stipe has a branchy structure and it becomes tough as it matures (Yeh et al., 2011).

*Grifola frondosa* have some biological activities such as antitumor (Masuda et al., 2006; Cui et al., 2013), anti-inflammation (Han and Cui, 2012; Su et al., 2020), immunomodulation (Adachi et al., 1994), antivirus (Gu et al., 2007; Zhao et al., 2016), antidiabetic (Postemsky and Curvetto, 2015), immune-enhancing, anti-hypertensive, hypoglycemia (Chen and Seviour, 2007) and antioxidation (Yeh et al., 2011). It also contributes to metabolic disorders treatment like non-alcoholic fatty liver disease. It also has potential in the treatment of hyperlipidemia as well as hyperglycemia. Picture of *Grifola* shown in Figure 8Bl.

### *Lentinus* sp.

The genus *Lentinus* belongs to the family Polyporaceae and it is mainly spread in the subtropical regions (Kirk et al., 2008). The species of *Lentinus* have extracellular enzymes, moreover, it acts as wood-decaying basidiomycetes, gregarious on fallen wood of a wide variety of deciduous trees like chestnut, cotton, hornbeam, beech, mulberry, ironwood, shii, oak, maple, chinquapin, sweetgum, and alder in a moist or warm climate (Bisen et al., 2010). Medicinally, *Lentinus edodes* is used for the diseases such as fungal infection, bronchial inflammation, hyperlipidemia, hepatitis, cancer, depressed immune function, heart disease, infectious disease, flu and cold, environmental allergies, urinary inconsistencies, hypertension, diabetes, etc. (Bisen et al., 2010). Picture of *Lentinus* shown in Figure 8Cm.

### *Pleurotus* sp.

*Pleurotus* is the genus of gilled mushroom and belongs to the family pleurotaceae. The species *Pleurotus ostreatus* also known as abalone or tree or oyster mushroom and is an edible mushroom (Chang and Miles, 2004). This mushroom is also used for mycoremediation and attacks and kills nematodes as well as microbes. This mushroom is mostly cultivated on straw and other media. This mushroom has the bitter-sweet aroma of benzaldehyde (Stamets, 2000b). This species of mushroom mainly grows on wood in shelflike clusters. This mushroom is of a larger size, and has white gills that run down a stubby. It has no stem. It has a white to lilac pattern of spores. This *Pleurotus ostreatus* has an oyster shaped, fan and broad cap spanning 5 to 25 cms. It is tan to dark-brown or white to gray in color. When it is young, its margin is enrolled and then becomes smooth and lobed or wavy. Because of its stipe arrangements, its flesh is firm, white, and of different thicknesses. The gills are white to cream in appearance (Philips and Roger, 2006). This mushroom has some biological effects due to the presence of some nutritional compositions including anti-cholestrolic, anticancer, antiviral, anti-diabetic, antioxidant, eye health, antibacterial and antiarthritic, etc. (Deepalakshmi and Mirunalini, 2014). Picture of *Pleurotus* shown in Figure 8Cn.

### *Calocybe* sp.

The genus *Calocybe* is the fungus that belongs to the family Lyophyllaceae. The species *Calocybe indica* is locally known as a milky-white mushroom that is rich in ascorbic acid, lipids, riboflavin, amino acids, pyridoxine, vitamins, biotin, low fat, nicotinic acid, proteins, minerals (arsenic, zinc, potassium, manganese, calcium, phosphorus, magnesium, iron and sodium), fibers. According to Mandal et al. (2012) this mushroom has antioxidant properties and stimulated immune activation of thymocytes, splenocytes, and macrophages.

It helps in reducing the triglycerides and total plasma cholesterol level and consequently decreases the chance of cardiovascular, artery, and atherosclerosis-related disorders (Alam et al., 2008).

It also possesses biological activities regarding neurodegenerative diseases, anti-carcinogenesis, anti-aging, anti-obesity, cardiovascular disease, anti-infection, preventing physical injury, anti-tumor also helps to reduce the risk of breast cancer (Tsukamoto et al., 2003; Sharma et al., 2013). Picture of *Calocybe* shown in Figure 8Co.

### *Huitlacoche* sp.

*Huitlacoche* is a heterothallic fungus that belongs to the family Ustilaginaceae. This fungus is also known as Mexican truffle, corn mushroom, or corn smut. This mushroom has great importance for the human diet (Valverde et al., 2012). This mushroom contains some bioactive compounds such as proteins, amino acids, glutamic acid, lysine, serine, aspartic acid, glycine, total carbohydrates, arabinose, mannose, galactose, xylose, glucitol, mannitol, glycerol, etc. Out of these, it has been found that xylose, galactose, mannose, and arabinose are present in lower proportions. It also contains heteroglycans, dietary fiber, and homoglycans, etc. (Valverde et al., 2012).

*Huitlacoche* mushrooms possess antitumoral, antimutagenic, immunomodulating, antiatherogenic, hyperlipidemic, hypo- glycemic, anti-inflammatory as well as various other health-promoting activities (Valdez-Morales et al., 2010). Picture of *Huitlacoche* shown in Figure 8Cp.

### *Cordyceps* sp.

*Cordyceps* are the fungus that mainly lives on certain caterpillars and are usually found in high mountain regions (Kindzel’skii et al., 1995). The species *Cordyceps sinensis* comprises cordycepic acid and cordycepin substances. This mushroom has some therapeutic applications such as stabilization of blood sugar metabolism, effects of enhanced utilization of oxygen, and production of ATP. This mushroom protects the organs from kidney, liver, and heart diseases. *Cordyceps sinensis* also has a sedative effect on the central nervous system of the body (Zhou et al., 2009).

This *Cordyceps* mushroom also has immunomodulating effects and also possesses anticancer activities, especially skin and lung cancer. It also shrinks the size of tumors (Kindzel’skii et al., 1995). Picture of *Cordyceps* shown in Figure 8Cq.

## Omics Approaches in Mushroom Research

There are several omics approaches in this post-genome era such as transcriptomics, metabonomics, genomics, and proteomics along with the bioinformatics that plas a vital role to assess the teratogenicity, nephrotoxicity, and genotoxicity of medicinal mushrooms. To predict the toxicity of chemicals, many genomes expression analyses have been performed in the form of metabolites, mRNA, and protein profiles that will have the same type of metabonomics, transcriptomics, and proteomics in a biological system such as toxic fingerprinting. Stierum et al. (2005), Xu et al. (2009), and Villeneuve and Garcia-Reyero (2011) demonstrated by *in silico* genome or comparative genomic analysis and elucidation of the inhibitory effect of toxic compounds, whether singly or in a mixture, can be used to discover the proteins or genes as an effective biomarker for the given toxicity (Stierum et al., 2005; Xu et al., 2009; Villeneuve and Garcia-Reyero, 2011).

Aardema and MacGregor suggested that the detailed study on metabonomics, transcriptomics, and proteomics profiles can be helpful in the exploration of the potential toxicity of any undesired mushroom compounds as well as their toxic outcomes (Aardema and MacGregor, 2002). Correspondingly, in the prediction of possible differential responses of several organisms that have various genomic compositions to several allergic as well as toxic compounds, toxicogenomics might also be helpful for several edible and non-edible mushrooms through *in vitro* as well as *in vivo* toxicity assessment.

## Current Status in the Mushroom Propagation

### Coriolus versicolor

Among all the mushrooms, *Coriolus versicolor* or *Trametes versicolor* is the best-studied and most potent medicinal mushroom. *Coriolus versicolor* has been well known for its anti-viral, anti-cancerous properties. This mushroom is mainly found in sub-tropical, tropicalm, and temperate zones and is highly adaptive. It has been found that the mycelial growth of medicinal mushroom *Coriolus versicolor* can be cultivated in the laboratory (Veena and Pandey, 2012).

### *Agaricus* sp.

Globally, *Agaricus* medicinal mushroom holds fourth place in commercial mushroom production and is commercially grown in at least 90 countries with more than 4 million tons estimated annual production (Sonnenberg et al., 2011; Royse, 2014). The cultivation of mycelial growth of medicinal mushroom *Agaricus* sp. can be cultivated in laboratories (Golmwen et al., 2019).

### *Ganoderma* sp.

The production of fruiting bodies of *Ganoderma* sp. is a long-term process that takes several months for the first fruiting body to even appear, depending upon the substrate as well as species. It has also been found that in submerged conditions, the growth of pure culture in liquid culture medium permits the acceleration of the growth due to which biomass can be brought to yield in several days (Asser et al., 2000).

It has been reported that the cultivation of medicinal mushroom *Ganoderma* sp. is possible in various labs in India. The mycelial of this medicinal mushroom grows well in potato dextrose medium at pH 5 and 25°C, followed by Kirk medium and glucose, malt extract as well as Kirk medium and molasses (Nasreen et al., 2005). Rai, Veena and Pandey, and Mishra and Singh, suggested that currently, medicinal mushrooms are gaining importance in the country (Rai et al., 2005; Veena and Pandey, 2012; Singh et al., 2014).

### *Hydnum* sp.

The cultivation of mycelial growth of medicinal mushroom *Hydnum* sp. can be cultivated in the laboratory. It has been found that the most suitable carbon and nitrogen sources for the mycelial growth of *Hydnum* sp. are glucose and mannitol (carbon sources) as well as Ca (NO_3_)_2_ (nitrogen source) whereas the poorest mycelial growth is found to be in xylose and sucrose (carbon sources) and (NH_4_)_2_HPO_4_ and NH_4_NO_3_ (nitrogen sources) (Peksen et al., 2013). According to Peksen et al. (2013), Peat and peat (vermiculite mixtures) are the best media that can be used for the production of vegetative inoculum of *Hydnum* sp. (Peksen et al., 2013).

### *Coprinus* sp.

It has been seen that the culture of medicinal mushroom *Coprinus* sp. can be cultivated in laboratories (Luo et al., 2004).

### *Morchella* sp.

The biomass production of medicinal mushroom *Morchella* sp. can be done in laboratories by using Malt extract agar medium (MEA). It has also been found that the mixture of peat, sawdust, and chestnut crust (domestic waste); peat; peat, rice hulls; peat, wheat; peat, potato crust (domestic waste); peat, sawdust and peat, rice hulls, sawdust can be used as additional material (Eliuz and Goksen, 2017).

### *Cantharellus* sp.

The cultivation of mycelial growth of medicinal mushroom *Cantharellus* sp. can be done in laboratories. It has been found that Murashige and Skoog (MS) medium can be used for the growth along with nitrogen (peptone, beef extract, ammonium citrate, potassium nitrate ammonium nitrate, yeast extract, sodium nitrate, and ammonium sulfate) and carbon (xylose, galactose, glucose, sucrose, and fructose) sources (Deshaware et al., 2021).

### *Amanita* sp.

The cultivation of mycelial growth of medicinal mushroom *Amanita* sp. can be done in the laboratory and has also been found that the species in subgenus *Lepidella* are more easily to isolate and grow in the culture as compared to the species of subgenus *Amanitopsis* (Zhang et al., 2005).

### *Cortinarius* sp.

Globally it has been found that the *Cortinarius* sp. is one of the largest genera of medicinal mushrooms. Although the genus *Cortinarius* is very diverse and it is very easy to figure out the species of *Cortinarius* from other species of mushroom because of the presence of diagnostic macroscopic characteristics (Itoo et al., 2015).

The cultivation of medicinal mushroom *Cortinarius* sp. can be achieved easily in a laboratory (Matheny et al., 2006).

### *Tremella* sp.

The mycelial growth of medicinal mushroom *Tremella sp.* can be cultured in laboratories. It has also been found that this mycelial growth of *Tremella* sp. can be grown on potato dextrose agar along with cycloheximide (Jang et al., 2014).

### *Rigidoporus* sp.

The cultivation of mycelial growth of medicinal mushroom *Rigidoporus* sp. can be achieved in laboratories. It has also been found that the mycelial growth of *Rigidoporus* sp. can be grown in potato dextrose agar medium and is amended with chloramphenicol and streptomycin sulfate for the suppression of bacterial growth (Go et al., 2021).

### *Grifola* sp.

The mycelial growth of medicinal mushroom *Grifola* sp. can be cultivated in labs. It has been demonstrated that the production of mycelial growth of *Grifola* sp. can occur in a basal nutrient medium (peptone, sucrose, CaCl_2_.2H_2_O, yeast extract, MnSO_4_.H_2_O, NH_4_NO_3_, KNO_3_, KPO_4_H_2_, H_3_BO_3_, KI, Na_2_MoO_4_.2H_2_O, ZnSO_4_.7H_2_O, MgSO_4_.7H_2_O) supplemented with amino acids (inositol, thiamine, pyridoxine, nicotinic acid); vitamins; ascorbic acid (Cicarelli, Argentina, Santa Fé); folic acid (alanine, riboflavin, phenylalanine and tryptophan) (Postemsky and Curvetto, 2015).

### *Lentinus* sp.

The cultivation of mycelial growth of *Lentinus* sp. works in the laboratory (Mossebo, 2002). It has been found that *Lentinus* sp. evidenced the growth of mycelia and found the high density in potato dextrose agar whereas a lesser density of mycelia was found in rice bran extract agar (Castillo et al., 2017).

### *Pleurotus* sp.

The fruiting bodies of medicinal mushroom *pleurotus* sp. can be produced in laboratories (Shnyreva et al., 2017). It has been found that the mycelial growth of *pleurotus* sp. can be done on potato dextrose agar (Silva et al., 2013).

### *Calocybe* sp.

The cultivation of the medicinal mushroom *Calocybe* sp. can work in laboratories. For the culture of medicinal mushroom *Calocybe* sp., potato dextrose agar medium can be used (Singh et al., 2017).

### *Huitlacoche* sp.

The mycelial growth of medicinal mushroom *Huitlacoche* sp. can be grown in laboratories (Montiel et al., 2015).

### *Cordyceps* sp.

The cultivation of the mycelial growth of medicinal mushroom *Cordyceps* sp. can be grown in culture in laboratories (Ghatnur et al., 2015).

## Conclusion

Mushrooms are a valuable fungus that occurs as an essential component of the ecosystem. Globally, mushrooms have been well known for their nutritional and therapeutic values. They are an economically, biotechnologically, and nutritionally valued group of organisms because of their importance in medicines, biocontrol, food, and the biological, chemical, cosmeceutical, and various other industries. Medicinal mushrooms have various bioactive (primary as well as secondary) metabolites that have been explored but there are still many indigenous mushrooms and their metabolites are unknown, therefore, it is necessary for researchers to discover many other unknown metabolites which are not discovered yet. Medicinal mushrooms also possess several essential biological effects that are beneficial for human health. Genomics, proteomics, and metabolomics can play a major role in improving the active metabolic component of mushroom research.

## Author Contributions

SKK created the manuscript idea and structure. AB and SKK wrote the manuscript. MS, VM, and SM took suggestions and discussions continuously for the improvement of the manuscript. All authors had gone through the manuscript and revised it before submission.

## Conflict of Interest

The authors declare that the research was conducted in the absence of any commercial or financial relationships that could be construed as a potential conflict of interest.

## Publisher’s Note

All claims expressed in this article are solely those of the authors and do not necessarily represent those of their affiliated organizations, or those of the publisher, the editors and the reviewers. Any product that may be evaluated in this article, or claim that may be made by its manufacturer, is not guaranteed or endorsed by the publisher.

## Abbreviations

ITS: Internal Transcribed Spacer
HPLC: high-pressure liquid chromatography
NMR: nuclear magnetic resonance spectroscopy microscopy
TLC: thin-layer chromatography
GC-MS: gas chromatography-mass spectrometry
UPLC: ultra-performance liquid chromatography
FTIR: fourier transform infrared spectroscopy
LCMS-TOF: liquid chromatography quadrupole time-of-flight mass spectrometry
HPTLC: high-performance thin-layer chromatography
MALDI-TOF MS: matrix-assisted laser desorption ionization-time of flight mass spectrometry
USDA: United States Department of Agriculture
NO: nitric oxide
HPV 16: anti-human papillomavirus 16
AChE: acetylcholinesterase
BChE: butyrylcholinesterase.
